# *Salvia miltiorrhiza* in thorax and abdomainal organ fibrosis: A review of its pharmacology

**DOI:** 10.3389/fphar.2022.999604

**Published:** 2022-09-20

**Authors:** Zhao Yang, Jingshu Qi, Dabing Ping, Xin Sun, Yanyan Tao, Chenghai Liu, Yuan Peng

**Affiliations:** ^1^ Institute of Liver Diseases, Shuguang Hospital Affiliated to Shanghai University of Traditional Chinese Medicine, Shanghai, China; ^2^ Shanghai Key Laboratory of Traditional Chinese Clinical Medicine, Shanghai, China; ^3^ Key Laboratory of Liver and Kidney Diseases, Ministry of Education, Shanghai, China

**Keywords:** organ fibrosis, *Salvia miltiorrhiza*, ingredients, pharmacological mechanism, review

## Abstract

Organ fibrosis is a common pathological change that finally results in organ failure, which involves the destruction of parenchyma cells, the activation of mesenchymal cells and the imbalance of immunological cells. In recent years, although some breakthroughs have been made in understanding the pathogenesis and therapeutics of organ fibrosis, no registered drugs could directly target the fibrotic process, which constitutes a major biomedical challenge. *Salvia miltiorrhiza* (SM) is a well-known medicinal plant in China, which has been widely applied because of its pharmacological effects on anti-oxidative, anti-myocardial infarction, anti-fibrotic, anti-inflammatory, and anti-neoplastic properties. Accumulated evidence suggested that SM played critical roles against organ fibrosis *in vivo* and *in vitro* experiments by its multiple biological compounds. In this review, we discussed the recent advances on the phytochemistry and pharmacological mechanisms of SM and its active ingredients in liver, lung, kidney, and heart fibrosis, which might help to promote the treatment of fibrotic diseases in thorax and abdomainal viscera in clinic.

## Introduction

Fibrosis, defined as fibroblast proliferation and excessive accumulation of extracellular matrix (ECM) in the broadest sense, was associated with a high cost in morbidity and mortality at a global level (Wynn and Ramalingam, 2012). In solid organ fibrosis, such as thorax and abdomainal organ fibrosis, activated fibroblasts presented overwhelming proliferating and invasion capacities, which could accelerate the development of fibrosis pathogenesis (Deng et al., 2021). Myofibroblasts, differentiated from fibroblasts, were then accumulated dramatically while ECMs were simultaneously synthesizd and deposited. Thus, these abnormal cell populations could contribute to the induction of fibrosis in major organs.

To date, many human diseases, including those of lung, heart, liver, kidney, bone marrow, brain blood vessels, and skin, correlated strongly with fibrosis. The main characteristics of organ fibrosis were typically presented with the chronic inflammation, the microvascular disturbances, the missing organ parenchyma and the loss-off function (Eddy, 2005). Therefore, fibrosis is a common pathway that might finally lead to organ failure. It was clear that organ fibrosis was a major clinical challenge. Currently, no registered drugs could directly target the fibrotic process. In contrast, traditional Chinese medicine (TCM) and its active ingredients had potential to target fibrosis in one organ or synchronously reversing fibrosis in multiple other fibrotic organs, which were increasingly recognized as effective therapies for fibrosis.

**FIGURE 1:**
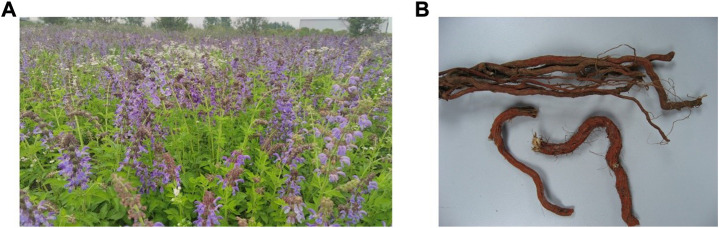
Overall appearance of *Salvia miltiorrhiza* Bunge (SM). **(A)**The aerial parts of SM. **(B)** The raw herb of SM.

Herbal medicine and its active ingredients were believed to treat disease as a trusted source of medicine from ancient times. *Salvia miltiorrhiza* (SM) Bunge (Lamiaceae), known as danshen (Chinese), is a widely used medicinal plant in TCM (Figure 1). It has been used in China with a long history of two thousand years, which was recorded in the oldest materia medica book “Shen Nongs Classic of Materia Medica” (Shen Nong Ben Cao Jing, 100 BCE to 200 CE). Historically, SM was used to promote blood circulation for removing blood stasis, improving microcirculation and assuaging pain. In addition, SM was demonstrated to exert numerous pharmacological effects, including anti-oxidative, myocardial infarction, anti-fibrotic (Su et al., 2015), anti-inflammatory (Ma et al., 2016), anti-hypertension (Lee et al., 2009), and anti-neoplastic (Chen et al., 2014) and anti-bacterial (Lee and Kim, 2016) properties.

**FIGURE 2:**
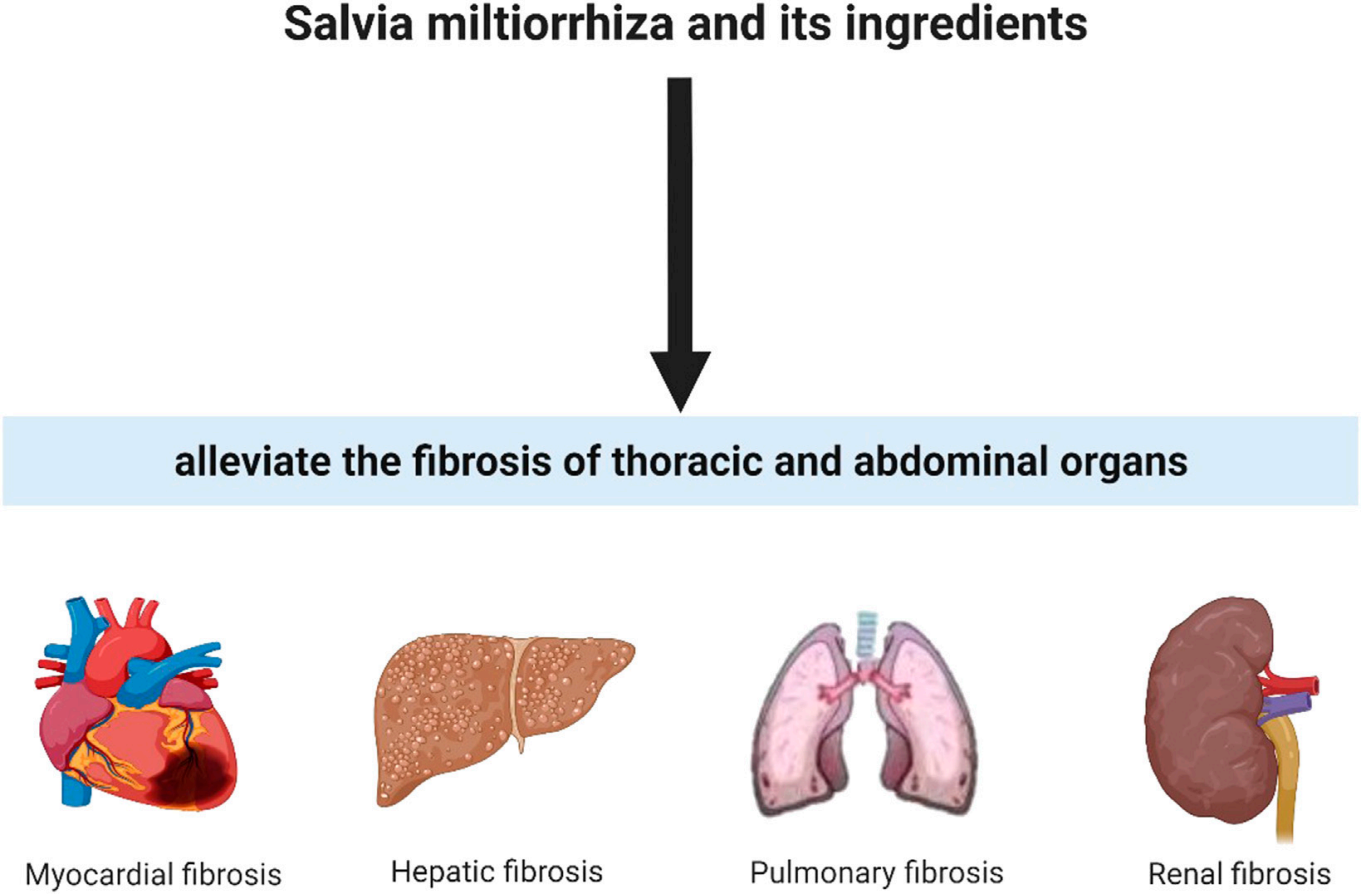
*Salvia miltiorrhiza* and its ingredients could alleviate fibrotic condition in thoracic and abdominal organs.

**FIGURE 3:**
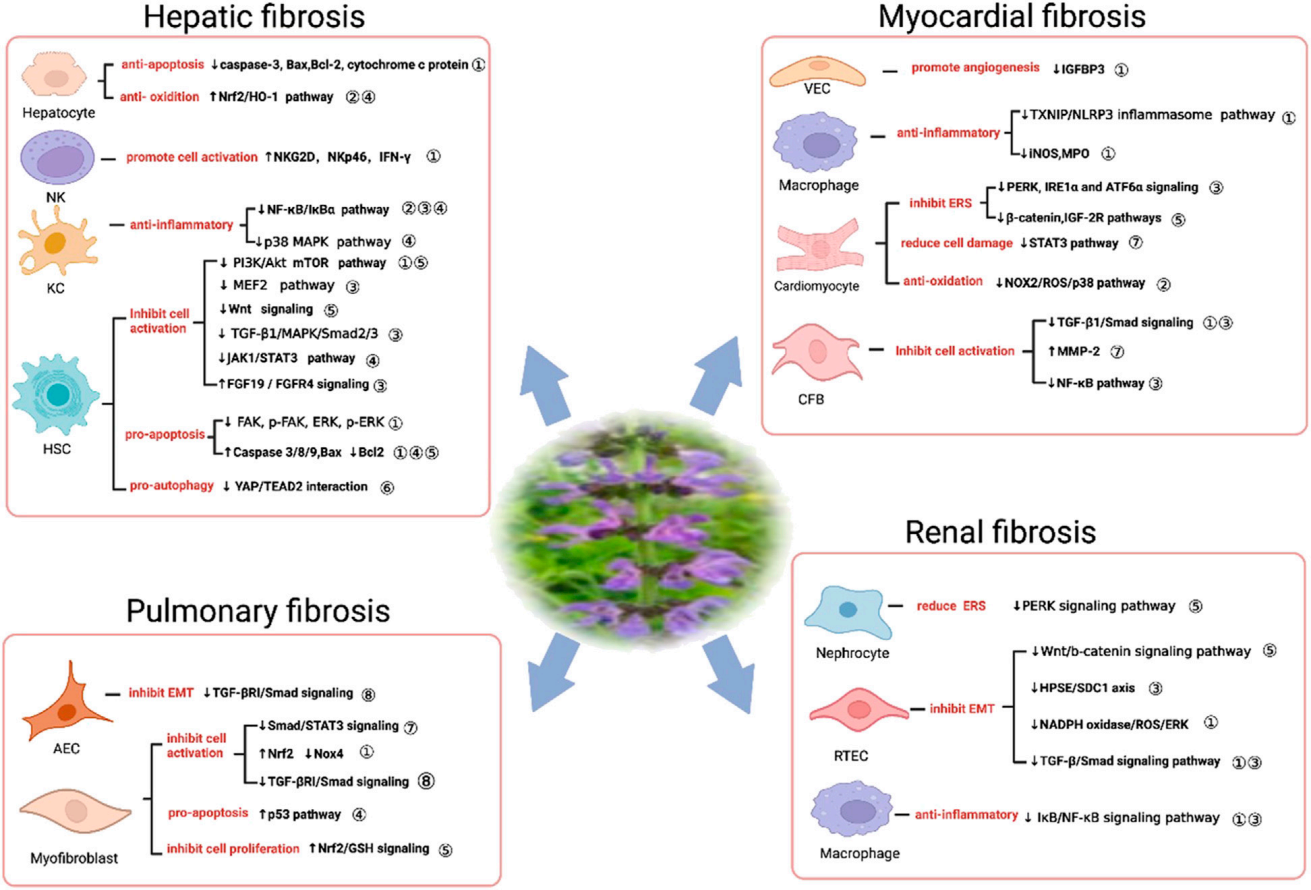
Pharmacological mechanisms of SM and its active ingredients in liver, lung, kidney and heart fibrosis. Ingredients in SM, ① *Salvia miltiorrhiza*; ② Tanshinol; ③ Salvianolic acid B; ④ Salvianolic acid A; ⑤ Tanshinone IIA; ⑥ Dihydrotanshinone I; ⑦ Cryptotanshinone; ⑧ Magnesium Lithospermate B. Abbreviations, NK, natural killer cells; KC, Kupffer cell; HSC, Hepatic stellate cell; VEC, vascular endothelial cell; CFB, myocardial fibroblast; AEC, alveolar epithelial cell; RTEC, renal tubular epithelial cell; Bax, B-cell lymphoma-2-associated X protein; BCL2, B-cell lymphoma-2; Nrf2, nuclear factor-erythroid 2-related factor 2; HO-1, heme oxygenase-1; NKG2D, natural killer cell group 2D; NF-κB, nuclear factor kappa-B; IκB, inhibitor of nuclear factor kappa-B; MAPK, mitogen-activated protein kinase; PI3K, phosphatidylinositol-3-kinase; Akt, protein kinase B; mTOR, molecular target of rapamycin; MEF2, myocyte enhancer factor 2; TGFβ1, transforming growth factor β1; Smad, *drosophila* mothers against decapentaplegic protein 2/3; JAK1, Janus kinase one; STAT3, signal transducer and activator of transcription three; FGF19, fibroblast growth factor 19; FGFR4, fibroblast growth factor receptor 4; FAK, focal adhesion kinase; p-FAK, phosphorylated focal adhesion kinase; ERK, extracellular regulated protein kinases; p-ERK, phosphorylated extracellular regulated protein kinases; YAP, yes-associated protein; TEAD2, TEA domain family member two; IGFBP3,insulin-like growth factor-binding protein 3; TXNIP, thioredoxin interacting protein; NLRP3, Nucleotide-binding oligomerization domain; leucine-rich repeat and pyrin domain-containing three; iNOS, inductible nitric oxide synthase; MPO, myeloperoxidase; PERK, protein kinase R-like endoplasmic reticulum kinase; IRE1α, Inositol requiring enzyme1α; ATF6α, activating transcription factor 6α; IGF-2R, insulin-like growth factor 2 receptor; NOX2, nicotinamide adenine dinucleotide phosphate oxidase two; ROS, reactive oxygen species; MMP-2, matrix metalloproteinase-2; EMT, epithelial-mesenchymal transition; TGF-βRI, transforming growth factor β receptor I; GSH, glutathione; ERS, endoplasmic reticulum stress; HPSE, heparinase; SDC1,syndecan-1; NADPH oxidase, reduced nicotinamide adenine dinucleotide phosphate oxidase; ↑ activate; ↓ inhibit).

*Salvia miltiorrhiza* Bunge contains ethanol-soluble compounds (such as various tanshinone analogues) and water-soluble active components (such as salvianolic acids) (Li et al., 2009; Pang et al., 2016). Accumulated evidence suggested that SM played critical roles against organ fibrosis in both animal experiments and clinical studies by its multiple biological ingredients, including anti-inflammation, anti-fibrosis, anti-oxidation and anti-apoptosis. In order to adequately define and elucidate the pharmacological functions of SM in organ fibrosis, pharmacology, phytochemistry, and safety of SM in organ fibrosis were hereby reviewed. For better understanding the pharmacological actions of SM against organ fibrosis, phytochemistry of SM were firstly summarized (Figures 2, 3).

## Phytochemistry of *Salvia miltiorrhiza*

There are more than 100 ingredients that were identified from SM so far, including lipid-soluble tanshinones and water-soluble phenolic compounds (Ma et al., 2015). Over 30 phenolic acids had been isolated from SM(Pang et al., 2016), including salvianolic acid A/B/C/D/E/F/G/H/I/J/K/N, rosmarinic acid, danshensu, protocatechuic acid, caffeic acid, lithospermic acid, caffeic acid, etc and other derivatives. Most of the phenolic acids had been conventionally isolated by continuous refluxing extraction and sonication in different solvents such as methanol, aqueous methanol (75%–95%, *v*/*v*) and aqueous ethanol (60%–95%, v/v) (XD et al., 2019). However, these methods also have some shortcomings. Most of them were time-consuming, which might promote phenolic acids converted into another kinds of compounds. In the last few years, novel emerging methods have been applied to extract phenolic acids, including microwave-assisted extraction (Gallo et al., 2010), supercritical fluid extraction (SFE) (Li et al., 2002), ultrasonic extraction (UAE) (Li et al., 2009), tissue-smashing based ultra-rapid extraction (Fan et al., 2014) and microsphere resin chromatography combined with microbial biotransformation (Kan et al., 2009).

Besides the hydrophilic salvianolic acids, the lipophilic terpenoids were also the major bioactive constituents of SM. Until now, at least 40 liposoluble compounds had been isolated from SM. Tanshinones and their analogues, including tanshinone I, tanshinone IIA, tanshinol A, tanshinol B, cryptotanshinone, dihydrotanshinone, danshenxinkun A, przewaquinone A, etc, were the main active diterpenoids in SM (Su et al., 2015). Some conventional extraction reagents, such as CHCl3, ethyl acetate, or petroleum ether, were used as the initial extraction solvent to isolate the tanshinones (XD et al., 2019). Besides these, extraction techniques such as soaking, percolation, reflux as well as ultrasound were generally applied for the extraction of tanshinones. However, tanshinones were present at lower concentrations than in the original SM, and many liposoluble constituents were instability, eg. cryptotanshinone and tanshinone IIA, etc (Li et al., 2008). Nowadays, these problems could be solved by a wide range of technique or approach, including infrared-assisted extraction (Chen et al., 2010), UAE (Li et al., 2009), surfactant-assisted extraction (Bi et al., 2011), SFE (Li et al., 2002) and pressurized-liquid extraction (Jiang et al., 2010).

Apart from the above, as SM was cultivation is scattered all over the country, the contents of main active constituents of SM might be influenced by environmental, altitude and cultivars factors (Wang et al., 2013; Zhao et al., 2016a). In addition, the active constituents might differ in intrinsically because of the various germplasms (Zhang et al., 2013; Zhao et al., 2016b). And the different genotypes of SM possessed their own specific ethylene responsive element binding protein gene (Cui et al., 2010).

## Pharmacological actions of *Salvia miltiorrhiza* in treatment for organ fibrosis

### Liver fibrosis

Liver fibrosis is a key pathological hallmark of various chronic liver diseases, including auto-immune liver disease, viral hepatitis, alcohol, and non-alcoholic fatty liver disease (Friedman, 2010; Tsochatzis et al., 2014). Although significant achievements have been made in our understanding of the pathogenenic actions of hepatic fibrosis and cirrhosis, effective anti-fibrotic agents and therapies remain the unconquered areas in the fields of drug research and development (Schuppan and Kim, 2013). “Deficiency of vital essential and blood stasis” was response in development of liver fibrosis and cirrhosis, which was the basic TCM pathogenesis of this condition. Among these, blood stasis was a central component. In TCM theory, SM was considered to promote blood circulation for removing blood stasis and resolving microcirculation (Sun et al., 2005). SM was applied in treating splenomegaly due to schistosomiasis in the fifties of the last century in China. Since then, many Chinese patent medicines, typified by Fuzheng Huayu tablet/capsule, contained SM as an integral part against liver fibrosis and cirrhosis. Both *in vitro* and *in vivo* experiments confirmed that SM played anti-fibrotic roles in liver fibrosis and cirrhosis (Peng et al., 2018). These hepatoprotective mechanisms of SM were shown to be attributed to inhibiting inflammation, reducing oxidant stress, enhancing apoptosis of HSCs, decreasing hepatocellular injury and suppressing the functions of activation of HSCs without damaging the hepatocytes. In addition to the direct effects of inhibiting the activated HCSs, SM could indirectly enhance the activities of NK cell to reduce liver fibrosis (Peng et al., 2018). Several experimental studies had reported that SM could couple with some herbal medicines, such as radix astragali (Yang et al., 2008; Cao et al., 2020), stephania tetrandra (Chor et al., 2009), ligusticum chuanxiong and glycyrrhiza glabra (Lin et al., 2008), to exert the antifibrotic effects *via* alleviating collagen deposition and reducing inflammation in the liver. Moreover, the active anti-fibrotic ingredients from SM have gained an increasing amount of attention. Salvianolic acid A/B/C, danshensu, tanshinone IIA and dihydrotanshinone Ⅰ were reported to have noticeable pharmacological activities and were also representative active ingredients in SM. The anti-fibrotic activities of these compounds were exhibited significantly, especially inhibiting the activation of HSCs which was a stromal cell in the liver well known for its role in triggering the fibrogenic process both *in vitro* and *in vivo*. These results indicated that SM alone or in combination with other herbs were highly effective in anti-fibrotic therapeutic strategy. And the inhibitory effect of the ingredients from SM might be a continuation of its anti-fibrotic effect. More details were shown in Table 1.

**TABLE 1:** Effects of SM and its active ingredients against liver fibrosis *in vivo* and *in vitro*.

Animals/Cells	Inducer	Drug and dose	Mechanism	References
***In vivo***				
C57BL/6 mice	10% CCl_4_ 2 ml/kg i.p	SM extract 3.0 g/kg i.g	NKG2D, Nkp46, IFN-γ↑	Peng et al. (2018)
SD rats	TAA 350 mg/kg i.p	PF2401-SF 1 or 2.5 mg/kg i.g	collagen 1(α), TIMP1, α-SMA↓	Parajuli et al. (2015)
SD rats	BDL	IH764-3 40 mg/kg i.p	α-SMA, FAK, p-FAK, ERK, p-ERK↓	Liu et al. (2012)
SD rats	50% CCl_4_ 1 ml/kg i.g	tanshinol 20 or 40 mg/kg i.g	SOD, GSH-Px, HO-1, NQO-1, GCLC, NF-κB, IκBα ↑	Wang et al. (2018b)
			HA, LN, IV-C, PIIIP, MDA, Cox-2, TGF-β, TNF-α, IL-1β, IL-6, NF-κB in the nucleolus↓	
SD rats	BDL	hot-water extract of SM 100 mg/kg i.g	TCHO, MDA, Hyp, α-SMA↓	Nan et al. (2001)
Kunming mice	0.1% DEN 10 ml/kg i.p	salvianolic acid B 10 or 30 mg/kg i.g	p-Smad3C↑	Wu et al. (2019)
			α-SMA,CollagenI, p-Smad2C, p-Smad2L,p-Smad3L↓	
SD rats	CCl_4_ 0.75 ml/kg i.g	PF2401-SF 50 mg/kg i.g	α-SMA↓	Parajuli et al. (2013)
Wistar rats	CCl_4_ 1 ml/kg i.p	Tanshinone IIA 10 mg/kg i.g	α-SMA, COL1A2, c-Jun, p-c-Jun, c-Myc, CCND1, MMP9, P65, p-P65, PI3K, P38↓	Shi et al. (2020)
Wistar rats	CCl_4_ 0.5 ml/kg i.p	SM extract 25 or 50 mg/kg i.g	GSH↑	Lee et al. (2003)
			GST, TGFβ1, TIMP-1, procollagen I↓	
SD rats	BDL	dihydrotanshinone I 25 mg/kg i.p	γ-GT, COL1A1¸ ACTA2, TGFβ1, MMP-2, TIMP-1, TIMP-2↓	Ge et al. (2017)
SD rats	CCl_4_	tanshinol 20 or 40 mg/kg i.g	MMP-13, MMP-1,Bax, Caspase- 3↑	Peng et al. (2017)
			PIIINP, HA, CollagenIV, LN, HOP, TIMP-1, Collagen I, Collagen II, α-SMA, TGFβ, Cox-2, TNF-α, IL-1, IL-6, Bcl-2, β-FGF, PD-ECGF↓, PI3K/AKT/mTOR/p70S6K1↓	
Wistar rats	CCl_4_ 0.5 ml kg i.p	water-soluble extract of SM 50 mg/kg i.g	GSH↑ caspase-3, Bax, Bcl-2, cytochrome c protein, calpain-µ↓	Lee et al. (2006)
SD rats	CCl_4_ 1 ml/kg i.g	salvianolic acid B 10 or 20 mg/kg i.g	NF-κB, IκBα in the cytoplasm↑	Wang et al. (2012)
			HA, LN, IV-C, PIIIP, NF-κB in the nucleolus↓	
***In vitro***				
JS-1 cell line	TGF-β1 5 ng/ml	SM extract 12.5–50 μg/ml	RAE-1ε↑	Peng et al. (2018)
			α-SMA↓	
T6 and LX-2 cell lines	TGF-β1 9p.m.	salvianolic acid B 25, 50 and 100 μM	p-ERK1/2, p-JNK1/2, p-P38, p-Smad2C, p-Smad2L, p-Smad3C, p-Smad3L, PAI-1↓	Wu et al. (2019)
LX-2 cell line	LPS 100 ng/ml	salvianolic acid B 1, 2.5 and 5 μM	FGF19, FGFR4 ↑	Tian et al. (2021)
			α-SMA, COL1A1↓	
t-HSC/Cl-6 cell line	Null	PF2401-SF 20 μg/ml	Caspase -3, Caspase -8, Caspase- 9, Bax↑	Parajuli et al. (2013)
			Bcl2↓	
LX-2 cell line	TGF-β1 2 ng/ml	dihydrotanshinone I 1, 5 and 10 μM	MAP1LC3B, LC3B↑	Ge et al. (2017)
			TGFβ1, α-SMA, COL1A1, pHSCs, ACTA2, CTGF, SOX4, p62↓	
LX-2 cell line	7-days culture	salvianolic acid B (6 μM, 48 μM), caffeic acid (6 μM, 48 μM) and rosmarinic acid (48 μM)	α-SMA↓	Yang et al. (2013)
human primary HSCs	TGF-β1 10 ng/ml	salvianolic acid B 1 μM	MEF2, α-SMA, Collagen I↓	Zhang et al. (2019a)
T6 cell line	acetaldehyde 200 μM	danshensu 100, 125 and 150 μM	uPA↑	Zhang et al. (2012)
			TGF-β1, PAI-1↓	
t-HSC/Cl-6 cell line	null	tanshinone IIA 20 μM	Caspase -3, cytochrome c, cyclin E, cyclin A, cdk2, Bax/Bcl-2↑	Che et al. (2010)
primary rat HSCs	TGF-β1 1 ng/ml	salvianolic acid B 280 μM	DPPH, MDA, ROS, α-SMA↓	Lin et al. (2006b)
T6 cell line	PDGF-BB	salvianolic acid A 10 mM	Caspase -3↑	Lin et al. (2006a)
			Bcl-2, p21, p27, Akt, cyclinsD1/E, PDGF↓	
primary rat HSCs	24-h culturing	salvianolic acid A 1 and 10 μM	Collagen I↓	Liu et al. (2000)

Note: i.p: intraperitoneal injection; i.g.: intragastric administration.

### Renal fibrosis

Renal fibrosis is a common feature of a range of chronic kidney diseases (CKDs) with the progressive and irreversible declines in renal functions. Renal tubulointerstitial fibrosis, glomerulosclerosis, and arteriosclerosis with perivascular fibrosis are the established characteristic of renal fibrosis (Liu, 2011). Excessive deposition of ECM, inflammatory cell infiltration, fibroblast accumulation, and myofibroblast expansion disrupt the local vasculature and impede the tissue repair, which accelerates the development of renal fibrosis in CKDs and eventually leads to kidney failure. Therefore, renal fibrosis is a hallmark of end-stage kidney disease.

Currently, despite remarkable progress in preclinical animal experiments, very limited therapeutics could inhibit or even reverse renal fibrosis effectively and safely. Haemodialysis, peritoneal dialysis and kidney transplantation are largely to the symptomatic approaches and palliation of symptoms, but cannot fundamentally improve the condition. In contrast, TCM can provide an alternative approach for treating renal fibrosis. SM and its main ingredients were demonstrated to have nephroprotective activities and anti-fibrotic functions via multiple pathways in adenine diet, streptozotocin (STZ) injection, 5/6 nephrectomy and unilateral urethral obstruction (UUO) induced renal fibrosis models (Table 2). Both water and ethanol extracts of SM presented protections in nephropathy and renal fibrosis *via* inhibiting the elevation of renal functions, improving the clinical symptoms of glomerular and tubular atrophy, alleviating focal calcium deposits, altering metabolites and reversing renal interstitial fibrosis and inflammation. Furthermore, it was revealed that SM suppressed renal fibrosis and epithelial trans-differentiation by inhibiting TGF-β/Smad and NADPH oxidase/ROS/ERK signaling pathways (Cai et al., 2018a). Beyond that, therapeutic application of SM was effective in combination with other agents. Astragalus membranaceus and SM could alleviate collagen deposition and metabolism, especially Tryptophan metabolism and Butanoate metabolism, in cyclosporin A-induced chronic nephrotoxicity and renal fibrosis. The further underlying mechanism might be lied in regulating the “gut-kidney axis” and modulating the disorder of miRNA-mRNA interaction profiles (Han et al., 2021).

**TABLE 2:** Effects of SM and its active ingredients against renal fibrosis *in vivo* and *in vitro*.

Animals/Cells	Inducer	Drug and dose	Mechanism	References
***In vivo***				
SD rats	adenine 150 mg/kg i.g	Ethanol extract of SM 0.46 g/kg i.g. and water extract of SM 1.03 g/kg i.g	UP, Scr, BUN, ISF, E-cadherin, α-SMA, FN, p-ERK, NOX1, NOX2, NOX4, TGF-β↓	Cai et al. (2018b)
C57BL/6 mice	UUO model	protocatechualdehyde (PCA) 10 or 40 mg/kg i.g	Smad7↑	Yang et al. (2021)
			KIM-1, BUN, SCR, α-SMA, collagenI, fibronectin, TNF-α, IL-1β, MCP-1, COX2, iNOS, NF-κB, Smad3↓	
C57BL/6 mice	UUO model	salvianolic acid B 6.25–25 mg/kg i.g	SDC1, E-cadherin↑	Hu et al. (2020)
			BUN, CR, HPSE, α-SMA, TGF-β1, FGF-2↓	
SD rats	streptozotocin 60 mg/kg i.p	tanshinone IIA 2, 4, 8 mg/kg i.p	SOD↑	Xu et al. (2020)
			TGF-β1, TSP-1, Grp78, CHOP, p-PERK, p-elf2α, ATF4 ↓	
SD rats	5/6 nephrectomy	tanshinone IIA 10 mg/kg i.g	Ang II, TGF-β1, collagen IV↓	Ahn et al. (2010)
SD rats	streptozotocin 60 mg/kg i.p	danshen injection 0.5–1 ml/kg i.p	SOD↑	Xu et al. (2016)
			ROS, MDA, TGF-β1,Smad2/3, TNF-α, IL-1β, IL-6, p-IκBα, p-NF-κB p65 ↓	
	streptozotocin 55 mg/kg i.p	danshen injection 0.78 ml/kg i.p	GSH-Px, SOD↑	Yin et al. (2014)
			AGEs, LPO, TGF-β1↓	
***In vitro***				
HK-2 cell line	ISF 250 μΜ	Ethanol or water extract of SM, 5–100 µM	α-SMA, FN, E-cadherin↑	Cai et al. (2018b)
			NOX1, NOX2, NOX4, p-ERK ↓	
			TGF-β, TGF-βRI, TGF-βRII, Smad2, Smad3, Smad7 ↓	
primary renal TECs	TGF-β1 2 ng/ml	protocatechualdehyde 20–80 μM	LRNA9884, iNOS, COX2, IL-6, MCP-1, NF-κB, IL-6, α-SMA, collagen I, fibronectin↓	Yang et al. (2021)
HK-2 cell line	AngII 1 µM	salvianolic acid B 0.1–10 µM	SDC1, E-cadherin↑	Hu et al. (2020)
			TGF-β1, FGF-2, HPSE, α-SMA↓	
HK-2 cell line	glucose 30 mM	Tanshinone IIA 5 or 10 μM	VDR, E-cadherin↑ a-SMA, b-catenin, GSK-3b↓	Zeng and Bao, (2021b)
HK-2 cell line	glucose 30 mM	Tanshinone IIA 1–50 μM	E-cadherin↑	Cao et al. (2017)
			α-SMA, vimentin, fibronectin, Snail↓	

In addition, active compounds in SM, such as protocatechualdehyde, salvianolic acid B, and tanshinone IIA, were also exert effects against renal fibrosis (Hu et al., 2020; Xu et al., 2020; Yang et al., 2021) in several renal fibrosis models. Protocatechualdehyde, a natural compound in the root of SM, was reported to decrease renal inflammation and fibrosis via mediating NF-κB/TGF-β1/Smad3/lncRNA9884/MCP-1 signaling pathway (Yang et al., 2021). Salvianolic acid B could notably reduce the renal injury and fibrosis in murine UUO model *in vivo*. The mechanism was confirmed to be related with the downregulation of HPSE/FGF-2/TGF-β1/α-SMA expression and upregulation of SDC1/E-cadherin levels *in vitro* (Hu et al., 2020). Tanshinone IIA was demonstrated to have excellent anti-fibrotic properties in streptozotocin-induced and 5/6 nephrectomy models, respectively (Ahn et al., 2010; Xu et al., 2020). More importantly, the mechanism for SM against renal fibrosis might be related to reducing endoplasmic reticulum stress to attenuate PERK signaling activities, decreasing expressions of Ang II, TGF-β1 and collagen IV, attenuating high glucose-induced EMT by up-regulating VDR levels on Wnt/β-catenin pathway and inhibiting HG-induced the epithelial-myofibroblast trans differentiation pathway (Ahn et al., 2010; Cao et al., 2017; Xu et al., 2020; Zeng and Bao, 2021a).

### Pulmonary fibrosis

Pulmonary fibrosis is a chronic interstitial pulmonary disease caused by a diversity of insults, including smoke, chemical materials, microbial infection, and environment contamination (Noble et al., 2012). Pulmonary fibrosis (rather difficult to reverse), consisting of progressive and irreversible destruction of lung architecture caused by scar formation, could ultimately lead to organ malfunction, disruption of gas exchange, and death from respiratory failure (Wynn, 2011). Till now, no effective therapy could prevent or reverse the development of pulmonary fibrosis. Nintedanib and pirfenidone are proved by FDA to alleviate lung function and lung fibrosis, however, neither of these drugs are able to reverse pulmonary fibrosis. Currently, the only life-saving treatment available for patients with progressive lung fibrosis is lung transplantation. Thus, identifying drugs to ameliorate the pulmonary fibrogenesis is urgently needed.

Recently, TCM has played an indispensable role in the treatment of pulmonary fibrosis *via* its multi-components, multi-targets and multi-pathways. SM and its ingredients were demonstrated to have effects in extenuating pulmonary fibrosis (Peng et al., 2019). The effect of SM for treatment in pingyangmycin-induced experimental model, was reported for the first time in 1987 in China (Chen, 1987). In Table 3, we summarized the available literatures related to the mechanisms of SM and its ingredients against pulmonary fibrosis during the past 35 years. Among these, bleomycin (BLM) and silica were commonly used to induce pulmonary fibrosis in rats and mice. And TGF-β1-stimulated cultured lung fibroblast, such as HLFs and MRC-5, exerted as an excellent model for evaluating anti-fibrotic compounds *in vitro*.

**TABLE 3:** Effects of SM and its active ingredients against pulmonary fibrosis *in vivo* and *in vitro*.

Animals/Cells	Inducer	Drug and dose	Mechanism	References
***In vivo***				
SD rats	intratracheal instillation of bleomycin 5 mg/kg	Cryptotanshinone 7.5–60 mg/kg	E-cadherin↑	Zhang et al. (2019b)
			Fibronectin, COL-I, COL-III, α-SMA, PAI-1, IL-6, TNF-α, p-STAT3Tyr705, p-STAT3Ser727↓	
C57BL/6 mice	intratracheal injection of bleomycin 1.25 U/kg	ethyl acetate extract of SM(EASM) 20, 40, 80 mg/kg	Nrf2↑	Peng et al. (2019)
			TGF-β, p-Smad3, α-SMA, Col-I, Nox4, acid-soluble collagen↓	
SD rats	intratracheal instillation of bleomycin 2 mg/kg	Magnesium Lithospermate B 50 mg/kg i.p	Col1A1, α-SMA, Col3A1, IL-4, IL-6, IL-13, TGF-β↓	Luo et al. (2021b)
Wistar rats	intratracheal injection of bleomycin 5 mg/kg	salvianolic acid A 2.5, 5, and 10 mg/kg i.v	TGF-β mRNA↓	Pan et al. (2014)
C57BL/6 mice	intratracheal injection of bleomycin 0.025U/mice	tanshinone IIA 5, 10, 20 mg/kg, i.g	Nrf2↑	An et al. (2019)
			Nox4, Smad3, TGF-β1, fibronectin, Col-I, Col-III, α-SMA↓	
SD rats	intratracheal instillation of bleomycin 3.5 U/kg	Salvianolic Acid B 10 mg/kg i.p	Col1a1, Col1a2, Ctgf, PAI-1, α-SMA↓	Liu et al. (2016)
Wistar rats	intratracheal injection of bleomycin 5 mg/kg	Salvianolic acid B 20 mg/kg i.v	GSH, Nrf2↑	Liu et al. (2018)
			α-SMA, MDA↓	

Human fetal lung fibroblasts (HLFs)	TGF-β1 5 ng/ml	Cryptotanshinone 1.5–6 mg/L	E-cadherin↑	Zhang et al. (2019b)
			Fibronectin, COL-I, COL-III, α-SMA, PAI-1↓	
			TGF-βR I, TGF-βR II, Smad2, Smad3 ↓	
Mice embryo fibroblasts (NIH-3T3)	TGF-β1 10 ng/ml	EASM 0.1, 1, 3 μg/ml	Nrf2↑	Peng et al. (2019)
			TGF-β1, Nox4, PKC-δ, p-Smad3, α-SMA↓	
Human lung fibroblasts (MRC-5)	TGF-β1 10 ng/ml	salvianolic acid B 20 μg/ml or sodium tanshinone ⅡA sulfonate 50 μg/ml	IL-1β, TNF-α, COL1A1, α-SMA, ACTA2↓	Jiang et al. (2020)
Human type II alveolar epithelial cell line (A549) or MRC-5 cell	TGF-β1 10 ng/ml	Magnesium Lithospermate B 30 or 50 μM	Col 1A1, Col 3A1, α-SMA↓	Luo et al. (2021b)
			TGF-βRI, Smad3 ↓	
Murine 3T6 fibroblasts	null	salvianolic acid A 6.25–25 μg/ml	p21, p53, caspase-3↑	Pan et al. (2014)
			cyclin D1, cyclin E1, cyclin B1, Bcl-2↓	
NIH-3T3	TGF-β1 10 ng/ml	tanshinone ⅡA 1–10 μM	Nrf2, GSH↑	An et al. (2019)
			Nox4, α-SMA, Col-I, Smad3, Col-III, PKCδ↓	
A549 cell line	TGF-β1 10 ng/ml and TNF-α 10 ng/ml	salvianolic acid B 50 μg/ml	CDH1↑	Liu et al. (2016)
			FN1, p-Smad3, p-ERK1/2, p-JNK↓	
MRC-5 cell line	TGF-β1 10 ng/ml	salvianolic acid B 40 μM	GSH, Nrf2↑	Liu et al. (2018)
			α-SMA, vimentin, fibronectin, ROS, MDA↓	

Peng et al. (2019) found that ethyl acetate extract of SM could alleviate bleomycin-induced lung fibrosis. The mechanism was associated with the reduction of ROS generation in fibroblasts, activation of Nrf2 pathway and inhibition TGF-β1/Smad3 pathway *in vivo* and *in vitro*. Single use of SM significantly ameliorated experimental lung fibrosis, and such effect was also exerted when combined with other herbal medicine. Combination of SM and ligustrazine were viewed to attenuate bleomycin-induced pulmonary fibrosis in rats *via* modulating TNF-α and TGF (Huang et al., 2018). Both the lipophilic ingredients (tanshinone IIA and cryptotanshinone) and hydrophilic ingredients (salvianolic acid A, salvianolic acid B, and magnesium lithospermate B) from SM have protective effects against pulmonary fibrosis, including reducing the proliferation of lung fibroblasts and protecting the alveolar epithelial integrity (Pan et al., 2014; Liu et al., 2018; Zhang Y. et al., 2019; Luo et al., 2021a).

Salvianolic acid B (SAB) was the most well studied active hydrophilic compound of SM against lung fibrosis. SAB had potent anti-fibrotic effects via blocking proliferation of lung fibroblasts, trans-differentiation and oxidative stress levels (Liu et al., 2018; Jiang et al., 2020). The pharmacological mechanisms of SAB were mainly related to the inhibition of TGF-βRI/Smad signaling in activated pulmonary fibroblasts. Tanshinone IIA were also weakened the myofibroblast proliferation by activating Nrf2/GSH signaling pathway to limit glutaminolysis (An et al., 2019).

### Myocardial fibrosis

Myocardial fibrosis (MF) is characterized by excessive deposition of ECM in the cardiac interstitium, which is a pathophysiologic component of many chronic myocardial diseases. Although the pathological processes of MF involved the complex interaction of multiple cell types, activated fibroblasts and myofibroblasts are the major contributors, serving as the main source of collagen fibres in cardiac fibrosis (Gonzalez et al., 2018). Clinically available drugs for treating MF were applied including angiotensin-converting enzyme inhibitors (lisinopril) (Brilla et al., 2000), type1 angiotensin Ⅱ-receptor antagonists (losartan) (Diez et al., 2002) and mineralocorticoid-receptor antagonists (spironolactone) (Izawa et al., 2005), which target renin-angiotensin-aldosterone system. Besides, loop diuretics (torasemide) were also applied in clinical practice that targeting extracellular collagen processing (Lopez et al., 2004). However, no specific drugs exist for reversing myocardial fibrosis to the present date.

SM has been used in Chinese folk medicine to treat heart diseases for a long history in China. In recent years, multiple SM preparations such as compound Danshen tablets, compound Danshen Dripping Pill, Danshen injections, Danshen tablets and Danshen capsules have been used in treatment of cardiovascular problems. According to Chinese medicine theory, SM is considered to promote blood circulation and alleviate blood stasis so as to relieve pain, eliminate blood stasis and promote blood flow in treatment of MF. Similar to PF, SM, and its ingredients (e.g., Salvanic acid B) restrained fibroblasts activation and inhibited collagen deposition through suppressing oxidative stress and TGF-β1/smad signaling pathway in MF, especially blocking cardiac fibroblast proliferation and ECM synthesis (Zhang et al., 2015; Qiu et al., 2018a; Gao et al., 2019a). The mechanisms were mainly associated with inhibiting TGF-β1 expression and Smad2/3 phosphorylation, as well as restraining the release of myeloperoxidase (MPO) (Wang et al., 2017). In addition, tanshinone IIA, the main lipophilic bioactive components of SM, reduced the Ang II-induced activation of β-catenin and IGF-2R pathways, apoptosis and cardiac remodeling via ERs (Chen et al., 2017a). More details were shown in Table 4.

**TABLE 4:** Effects of SM and its active ingredients against myocardial fibrosis *in vivo* and *in vitro*.

Animals/Cells	Inducer	Drug and dose	Mechanism	References
*In vivo*				
Kunming mice	Iron Dextran Injection 50 mg/kg i.p	SM injection 3 g/kg and 6 g/kg i.p	SOD↑	Zhang et al. (2015)
			Hyp,MDA,COL-I,COL-III,TGF-β1, MMP-9↓	
C57BL/6J mice	Streptozotocin (STZ) 60 mg/kg i.p	Salvianolic acid B (Sal B) 15 or 30 mg/kg i.p	VEGFA, VEGFR2, p-AKT, p-ERK↑	Li et al. (2020)
			Collagen I, Collagen Ⅲ, IGFBP3↓	
Kunming mice	Isoproterenol hydro-chloride injection (ISO) 2.5 mg/kg i.p	Salvanic acid B	Smad7↑	Gao et al. (2019b)
			TGF-β1, Smad2/3↓	
SD rats	left anterior descending (LAD) ligation	Danshen Injection (DSI) 1.5 ml/kg/d i.m	Bcl-2, Bax↑	Wang et al. (2017)
			MMP-2, iNOS, MPO↓	
SD rats	left aortic descending coronary artery ligation	Cryptotanshinone (CTS) 30 and 60 mg/kg i.g	FN, COX-2, NOX-2,NOX-4↓	Ma et al. (2014)
SD rats	left anterior descending coronary artery ligation	Salvianolate 10, 20 and 40 mg/kg i.p	TGFβ1, p-Smad2/Smad2, p-Smad3/Smad3, Collagen I, Collagen III, MMP9, TIMP1, TXNIP, IL-1β, IL-18, NLRP3, Caspase-1, CRP, IL-6, BNP↓	Qiu et al. (2018b)
SD rats	left and right renal artery ligation	Tanshinone II-A 35 and 70 mg/kg i.g	MMP-9, TIMP-1, TIMP-2↓	Fang et al. (2010)
Wistar rats	Streptozotocin 65 mg/kg i.v	Cryptotanshinone 10 mg/kg i.g	STAT3, CTGF, MMP-9↓	Lo et al. (2017)
SD rats	isoprenaline 0.25 mg/kg i.p	isopropyl 3-(3,4-dihydroxyphenyl)-2-hydroxylpropanoate (IDHP) 50 mg/kg	Collagen I, Collagen III↓	Yin et al. (2015)
C57BL/6 mice	Isoprenaline 3 mg/kg s.c	Cryptotanshinone 20 mg/kg i.g	MMP-2↑	Ma et al. (2012)
*In vitro*				
Rat embryonic ventricular H9c2 cardiomyocytes	oxygen-glucose deprivation/reoxygenation (OGD/R) condition	PCA 1.25, 2.5 and 5.0 μM	Caspase-3, Bax↓	Wan et al. (2021)
			CHOP, BiP, PERK, Ero1-Lα, IRE1α, ATF6, HIF-1α↓	
Mouse cardiac fibroblasts (CFs) cells	TGF-β1 20 ng/ml	Sal B 5, 10, and 20 ng/ml	Smad7↑	Gao et al. (2019b)
			Smad2/3, MMP-2, MMP-9↓	
primary rat cardiac fibroblasts (CFs)	Ang II 100 nM	Cryptotanshinone (CTS) 2.5–20 mM	FN, CTGF, p-ERK1/2, ROS, NOX-2, NOX-4, COX-2↓	Ma et al. (2014)
primary neonatal rat cardiac fibroblasts	Ang II 1 μM	Salvianolic acid B (SalB) 12.5–50 μM	Collagen I, FN, CTGF, p-IκB, p-p65, α-SMA↓	Wang et al. (2018a)
Primary neonatal rat cardiomyocytes	d-glucose 30 mM	cryptotanshinone 3 μM	STAT3, CTGF, MMP-9↓	Lo et al. (2017)
neonatal rat cardiac fibroblasts (NRCFs)	isoprenaline	isopropyl 3-(3,4-dihydroxyphenyl)-2-hydroxylpropanoate (IDHP) 1–100 μM	ROS, p-p38, NOX2↓	Yin et al. (2015)
H9c2 cardiomyoblast cell	AngII 10^−8^M	Tanshinone IIA 40 μM	ERα, ERβ↑	Chen et al. (2017b)
			β-catenin, p-ERK1/2, IGF-2R, LEF-1, MMP-9, MMP-2, TGF-β1, p-Smad2/3, SP-1,CTGF↓	
Primary cardiac myocytes and cardiac fibroblasts from neonatal rats	endothelin-1 (ET-1) 10–8 M, phenylephrine (PE) 10–6 M, or insulin-like growth factor-1 (IGF-1) 10^–8^ M	tanshinone VI (tsh) 10^–5^ M	ET-1, PE, IGF-1↓	Maki et al. (2002)

## Conclusions and outlooks

Organ fibrosis was a common endpoint of diverse chronic diseases with progressive tissue scarring and organ dysfunction that eventually led to organ failure and significant mortality world-wide (Wynn, 2004). Pulmonary fibrosis, cardiac fibrosis, hepatic fibrosis, and renal fibrosis were the most common four types of organ fibrosis in thorax and abdomainal viscera, which shared the same histopathological features, including the destruction of parenchyma cells, the activation of mesenchymal cells, and the imbalance of immunological cells. Fibrosis is a highly dynamic process in various organ systems. Indeed, despite certain achievements were made in clinic treatment, no specific drug for reversing fibrosis of either organ was approved by Food and Drug Administration. Numerous anti-fibrotic drugs against single-target and single-pathway single target have failed in clinical experiments, which revealed that the candidate drug against organ fibrosis should shift to multi-target and multi-pathway.

SM has been regarded as the most frequent used hepatoprotective and cardioprotective drug in TCM practice. Accumulated evidence suggests that SM and its active ingredients exerted protective effects on fibrotic diseases in thorax and abdomainal viscera. The mechanism of how SM and its ingredients benefit fibrosis treatment including but not limited to decreasing inflammation, alleviating oxidative stress, regulating collagen production and degradation, and preventing tissue injury through different signaling pathways (Figures 2, 3). In fibrotic diseases, SM and its ingredients exerted anti-fibrotic functions in different organs via different mechanisms. But they share the same core aim: to lower the fibrous septa in the viscera. It has been known that the activated fibroblasts and myofibroblasts, mainly activated by TGF-β1, are the principal cells of producing ECM (Henderson et al., 2020). On the one hand, SM and its ingredients could inhibit the activation of fibroblasts and myofibroblasts through TGF-β/Smad signaling pathway in fibrotic organs, which contributed the acceleration of ECM degradation, decrease of collagen cross-linking and inhibition of collagen/ECM deposition. And on the other hand, ECM degradation, blocked by SM and its ingredients, could alleviate the cell-ECM interactions to limit the excessive activation of fibroblasts and myofibroblasts.

However, despite of the encouraging progress in our understanding of the efficacy of SM in organ fibrosis, a nonnegligible translational gap remained between bioactive novel constituents extracted form SM and conversion into effective patient therapies and pharmacological agents. Besides, most of the known mechanisms were explored from *in vitro* experiments with a single cell line. Some advanced experimental designs, such as 3D culture system of co-culturing with a diverse array of cells *in vitro* and transgenic mice experiments *in vivo*, were urgent needed to verity the above discoveries. And although the efficacy of Chinese patent medicine from SM and its ingredients have been repeatedly tested in clinical treatment of organ fibrosis, more further studies are needed to better understand the mechanism and to serve the patients. In addition, because of the insufficient bioavailability of SM and its ingredients in poor solubleness, which leads to low oral bioavailability and delivery problems, advanced drug carriers are meaningful to develop, so as to enhance the tissue targeting, expand the clinical applications for the patients suffering from organ fibrosis.

In summary, though many shortcomings exist in the current studies, pharmacological studies and clinical practices have demonstrated that SM and its ingredients are considered to have good clinical efficacy and widespread application prospects. With the implementations of further research, it is believed that more systematic molecular mechanisms and anti-fibrotic targets of SM and its ingredients could be identified and elucidated to improve the treatment for patients with organ fibrosis.

## References

[B1] Ahn Y. M. Kim S. K. Lee S. H. Ahn S. Y. Kang S. W. Chung J. H. (2010). Renoprotective effect of Tanshinone IIA, an active component of Salvia miltiorrhiza, on rats with chronic kidney disease. Phytother. Res. 24, 1886–1892. 10.1002/ptr.3347 21043035

[B2] An L. Peng L. Y. Sun N. Y. Yang Y. L. Zhang X. W. Li B. (2019). Tanshinone IIA activates nuclear factor-erythroid 2-related factor 2 to restrain pulmonary fibrosis via regulation of redox homeostasis and glutaminolysis. Antioxid. Redox Signal. 30, 1831–1848. 10.1089/ars.2018.7569 30105924

[B3] Bi W. Tian M. Row K. H. (2011). Extraction and concentration of tanshinones in *Salvia miltiorrhiza* Bunge by task-specific non-ionic surfactant assistance. Food Chem. 126, 1985–1990. 10.1016/j.foodchem.2010.12.059 25213987

[B4] Brilla C. G. Funck R. C. Rupp H. (2000). Lisinopril-mediated regression of myocardial fibrosis in patients with hypertensive heart disease. Circulation 102, 1388–1393. 10.1161/01.cir.102.12.1388 10993857

[B5] Cai H. Su S. Li Y. Zeng H. Zhu Z. Guo J. (2018b). Protective effects of Salvia miltiorrhiza on adenine-induced chronic renal failure by regulating the metabolic profiling and modulating the NADPH oxidase/ROS/ERK and TGF-β/Smad signaling pathways. J. Ethnopharmacol. 212, 153–165. 10.1016/j.jep.2017.09.021 29032117

[B6] Cai H. Su S. Li Y. Zeng H. Zhu Z. Guo J. (2018a). Protective effects of Salvia miltiorrhiza on adenine-induced chronic renal failure by regulating the metabolic profiling and modulating the NADPH oxidase/ROS/ERK and TGF-β/Smad signaling pathways. J. Ethnopharmacol. 212, 153–165. 10.1016/j.jep.2017.09.021 29032117

[B7] Cao L. Huang B. Fu X. Yang J. Lin Y. Lin F. (2017). Effects of tanshinone IIA on the regulation of renal proximal tubular fibrosis. Mol. Med. Rep. 15, 4247–4252. 10.3892/mmr.2017.6498 28440499

[B8] Cao T. Lu Y. Zhu M. Cheng J. Ye B. Fang N. (2020). Effects of Salvia miltiorrhiza and Radix astragali on the TGF-Î²/Smad/Wnt pathway and the pathological process of liver fibrosis in rats. Cell. Mol. Biol. 66, 46–51. 10.14715/cmb/2020.66.6.9 33040784

[B9] Che X. H. Park E. J. Zhao Y. Z. Kim W. H. Sohn D. H. (2010). Tanshinone II A induces apoptosis and S phase cell cycle arrest in activated rat hepatic stellate cells. Basic Clin. Pharmacol. Toxicol. 106, 30–37. 10.1111/j.1742-7843.2009.00465.x 19906051

[B10] Chen X. Guo J. Bao J. Lu J. Wang Y. (2014). The anticancer properties of salvia miltiorrhiza Bunge (danshen): A systematic review. Med. Res. Rev. 34, 768–794. 10.1002/med.21304 24123144

[B11] Chen X. Y. Yin W. P. Yin Q. Z. (1987). The dorsal raphe nucleus is involved in the inhibitory effect of hypothalamic arcuate stimulation on pain-evoked unit discharges of the thalamic parafascicular nucleus. Sheng Li Xue Bao 12, 46–53. 3603063

[B12] Chen Y. Duan G. Xie M. Chen B. Li Y. (2010). Infrared-assisted extraction coupled with high-performance liquid chromatography for simultaneous determination of eight active compounds in Radix Salviae miltiorrhizae. J. Sep. Sci. 33, 2888–2897. 10.1002/jssc.201000234 20730830

[B13] Chen Y. F. Day C. H. Lee N. H. Chen Y. F. Yang J. J. Lin C. H. (2017a). Tanshinone IIA inhibits beta-catenin nuclear translocation and IGF-2R activation via estrogen receptors to suppress angiotensin II-induced H9c2 cardiomyoblast cell apoptosis. Int. J. Med. Sci. 14, 1284–1291. 10.7150/ijms.20396 29104486 PMC5666563

[B14] Chen Y. F. Day C. H. Lee N. H. Chen Y. F. Yang J. J. Lin C. H. (2017b). Tanshinone IIA inhibits β-catenin nuclear translocation and IGF-2R activation via estrogen receptors to suppress angiotensin II-induced H9c2 cardiomyoblast cell apoptosis. Int. J. Med. Sci. 14, 1284–1291. 10.7150/ijms.20396 29104486 PMC5666563

[B15] Chor J. S. Yu J. Chan K. K. Go Y. Y. Sung J. J. (2009). Stephania tetrandra prevents and regresses liver fibrosis induced by carbon tetrachloride in rats. J. Gastroenterol. Hepatol. 24, 853–859. 10.1111/j.1440-1746.2008.05720.x 19220659

[B16] Cui G. H. Feng H. Li W. Y. Wang W. Y. Huang L. Q. (2010). Cloning and polymorphism analysis of SmERF in Salvia miltiorrhiza. Yao Xue Xue Bao 45, 1188–1193. 21351578

[B17] Deng C. C. Hu Y. F. Zhu D. H. Cheng Q. Gu J. J. Feng Q. L. (2021). Single-cell RNA-seq reveals fibroblast heterogeneity and increased mesenchymal fibroblasts in human fibrotic skin diseases. Nat. Commun. 12, 3709. 10.1038/s41467-021-24110-y 34140509 PMC8211847

[B18] Diez J. Querejeta R. Lopez B. Gonzalez A. Larman M. Martinez Ubago J. L. (2002). Losartan-dependent regression of myocardial fibrosis is associated with reduction of left ventricular chamber stiffness in hypertensive patients. Circulation 105, 2512–2517. 10.1161/01.cir.0000017264.66561.3d 12034658

[B19] Eddy A. A. (2005). Progression in chronic kidney disease. Adv. Chronic Kidney Dis. 12, 353–365. 10.1053/j.ackd.2005.07.011 16198274

[B20] Fan Y. Yan C. P. Chen C. So K. F. Li P. Qi L. W. (2014). Tissue-smashing based ultra-rapid extraction of chemical constituents in herbal medicines. J. Pharm. Biomed. Anal. 95, 213–219. 10.1016/j.jpba.2014.03.004 24685727

[B21] Fang J. Xu S. W. Wang P. Tang F. T. Zhou S. G. Gao J. (2010). Tanshinone II-A attenuates cardiac fibrosis and modulates collagen metabolism in rats with renovascular hypertension. Phytomedicine 18, 58–64. 10.1016/j.phymed.2010.06.002 20638255

[B22] Friedman S. L. (2010). Evolving challenges in hepatic fibrosis. Nat. Rev. Gastroenterol. Hepatol. 7, 425–436. 10.1038/nrgastro.2010.97 20585339

[B23] Gallo M. Ferracane R. Graziani G. Ritieni A. Fogliano V. (2010). Microwave assisted extraction of phenolic compounds from four different spices. Molecules 15, 6365–6374. 10.3390/molecules15096365 20877228 PMC6257672

[B24] Gao H. Bo Z. Wang Q. Luo L. Zhu H. Ren Y. (2019b). Salvanic acid B inhibits myocardial fibrosis through regulating TGF-β1/Smad signaling pathway. Biomed. Pharmacother. 110, 685–691. 10.1016/j.biopha.2018.11.098 30553195

[B25] Gao H. Bo Z. Wang Q. Luo L. Zhu H. Ren Y. (2019a). Salvanic acid B inhibits myocardial fibrosis through regulating TGF-β1/Smad signaling pathway. Biomed. Pharmacother. 110, 685–691. 10.1016/j.biopha.2018.11.098 30553195

[B26] Ge M. Liu H. Zhang Y. Li N. Zhao S. Zhao W. (2017). The anti-hepatic fibrosis effects of dihydrotanshinone I are mediated by disrupting the yes-associated protein and transcriptional enhancer factor D2 complex and stimulating autophagy. Br. J. Pharmacol. 174, 1147–1160. 10.1111/bph.13766 28257144 PMC5406384

[B27] Gonzalez A. Schelbert E. B. Diez J. Butler J. (2018). Myocardial interstitial fibrosis in heart failure: Biological and translational perspectives. J. Am. Coll. Cardiol. 71, 1696–1706. 10.1016/j.jacc.2018.02.021 29650126

[B28] Han C. Jiang Y. H. Li W. Liu Y. (2021). Astragalus membranaceus and Salvia miltiorrhiza ameliorates cyclosporin A-induced chronic nephrotoxicity through the "gut-kidney axis. J. Ethnopharmacol. 269, 113768. 10.1016/j.jep.2020.113768 33383113

[B29] Henderson N. C. Rieder F. Wynn T. A. (2020). Fibrosis: From mechanisms to medicines. Nature 587, 555–566. 10.1038/s41586-020-2938-9 33239795 PMC8034822

[B30] Hu Y. Wang M. Pan Y. Li Q. Xu L. (2020). Salvianolic acid B attenuates renal interstitial fibrosis by regulating the HPSE/SDC1 axis. Mol. Med. Rep. 22, 1325–1334. 10.3892/mmr.2020.11229 32626974 PMC7339410

[B31] Huang C. Wu X. Wang S. Wang W. Guo F. Chen Y. (2018). Combination of Salvia miltiorrhiza and ligustrazine attenuates bleomycin-induced pulmonary fibrosis in rats via modulating TNF-α and TGF-β. Chin. Med. 13, 36. 10.1186/s13020-018-0194-9 29997685 PMC6032559

[B32] Izawa H. Murohara T. Nagata K. Isobe S. Asano H. Amano T. (2005). Mineralocorticoid receptor antagonism ameliorates left ventricular diastolic dysfunction and myocardial fibrosis in mildly symptomatic patients with idiopathic dilated cardiomyopathy: A pilot study. Circulation 112, 2940–2945. 10.1161/CIRCULATIONAHA.105.571653 16275882

[B33] Jiang L. Wang J. Ju J. Dai J. (2020). Salvianolic acid B and sodium tanshinone II A sulfonate prevent pulmonary fibrosis through anti-inflammatory and anti-fibrotic process. Eur. J. Pharmacol. 883, 173352. 10.1016/j.ejphar.2020.173352 32645333

[B34] Jiang Y. David B. Tu P. Barbin Y. (2010). Recent analytical approaches in quality control of traditional Chinese medicines--a review. Anal. Chim. Acta 657, 9–18. 10.1016/j.aca.2009.10.024 19951752

[B35] Kan S. Li J. Huang W. Shao L. Chen D. (2009). Microsphere resin chromatography combined with microbial biotransformation for the separation and purification of salvianolic acid B in aqueous extract of roots of Salvia multiorrihza Bunge. J. Chromatogr. A 1216, 3881–3886. 10.1016/j.chroma.2009.02.084 19296955

[B36] Lee H. S. Kim Y. (2016). Antifungal activity of salvia miltiorrhiza against Candida albicans is associated with the alteration of membrane permeability and (1, 3)-beta-D-Glucan synthase activity. J. Microbiol. Biotechnol. 26, 610–617. 10.4014/jmb.1511.11009 26699747

[B37] Lee S. J. Park W. H. Moon H. I. (2009). Bioassay-guided isolation of antiplasmodial anacardic acids derivatives from the whole plants of Viola websteri Hemsl. Parasitol. Res. 104, 463–466. 10.1007/s00436-008-1205-z 18830630

[B38] Lee T. Y. Chang H. H. Wang G. J. Chiu J. H. Yang Y. Y. Lin H. C. (2006). Water-soluble extract of Salvia miltiorrhiza ameliorates carbon tetrachloride-mediated hepatic apoptosis in rats. J. Pharm. Pharmacol. 58, 659–665. 10.1211/jpp.58.5.0011 16640835

[B39] Lee T. Y. Wang G. J. Chiu J. H. Lin H. C. (2003). Long-term administration of Salvia miltiorrhiza ameliorates carbon tetrachloride-induced hepatic fibrosis in rats. J. Pharm. Pharmacol. 55, 1561–1568. 10.1211/0022357022098 14713368

[B40] Li C. L. Liu B. Wang Z. Y. Xie F. Qiao W. Cheng J. (2020). Salvianolic acid B improves myocardial function in diabetic cardiomyopathy by suppressing IGFBP3. J. Mol. Cell. Cardiol. 139, 98–112. 10.1016/j.yjmcc.2020.01.009 31982427

[B41] Li M. H. Chen J. M. Peng Y. Wu Q. Xiao P. G. (2008). Investigation of Danshen and related medicinal plants in China. J. Ethnopharmacol. 120, 419–426. 10.1016/j.jep.2008.09.013 18930799

[B42] Li Y. C. Zeng J. Q. Liu L. M. Jin X. S. (2002). Extraction of three tanshinones from the root of Salvia miltiorrhiza Bunge by supercritical carbon dioxide fluid and their analysis with high performance liquid chromatography. Se Pu 20, 40–42. 12541616

[B43] Li Y. G. Song L. Liu M. Hu Z. B. Wang Z. T. (2009). Advancement in analysis of Salviae miltiorrhizae Radix et Rhizoma (Danshen). J. Chromatogr. A 1216, 1941–1953. 10.1016/j.chroma.2008.12.032 19159889

[B44] Lin Y. L. Hsu Y. C. Chiu Y. T. Huang Y. T. (2008). Antifibrotic effects of a herbal combination regimen on hepatic fibrotic rats. Phytother. Res. 22, 69–76. 10.1002/ptr.2265 17724770

[B45] Lin Y. L. Lee T. F. Huang Y. J. Huang Y. T. (2006a). Antiproliferative effect of salvianolic acid A on rat hepatic stellate cells. J. Pharm. Pharmacol. 58, 933–939. 10.1211/jpp.58.7.0008 16805953

[B46] Lin Y. L. Wu C. H. Luo M. H. Huang Y. J. Wang C. N. Shiao M. S. (2006b). *In vitro* protective effects of salvianolic acid B on primary hepatocytes and hepatic stellate cells. J. Ethnopharmacol. 105, 215–222. 10.1016/j.jep.2005.10.021 16314058

[B47] Liu C. H. Liu P. Hu Y. Y. Xu L. M. Tan Y. Z. Wang Z. N. (2000). Effects of salvianolic acid-A on rat hepatic stellate cell proliferation and collagen production in culture. Acta Pharmacol. Sin. 21, 721–726. 11501181

[B48] Liu L. Wei J. Huo X. Fang S. Yao D. Gao J. (2012). The Salvia miltiorrhiza monomer IH764-3 induces apoptosis of hepatic stellate cells *in vivo* in a bile duct ligation-induced model of liver fibrosis. Mol. Med. Rep. 6, 1231–1238. 10.3892/mmr.2012.1076 22971838

[B49] Liu M. Xu H. Zhang L. Zhang C. Yang L. Ma E. (2018). Salvianolic acid B inhibits myofibroblast transdifferentiation in experimental pulmonary fibrosis via the up-regulation of Nrf2. Biochem. Biophys. Res. Commun. 495, 325–331. 10.1016/j.bbrc.2017.11.014 29108993

[B50] Liu Q. Chu H. Ma Y. Wu T. Qian F. Ren X. (2016). Salvianolic acid B attenuates experimental pulmonary fibrosis through inhibition of the TGF-β signaling pathway. Sci. Rep. 6, 27610. 10.1038/srep27610 27278104 PMC4899783

[B51] Liu Y. (2011). Cellular and molecular mechanisms of renal fibrosis. Nat. Rev. Nephrol. 7, 684–696. 10.1038/nrneph.2011.149 22009250 PMC4520424

[B52] Lo S. H. Hsu C. T. Niu H. S. Niu C. S. Cheng J. T. Chen Z. C. (2017). Cryptotanshinone inhibits STAT3 signaling to alleviate cardiac fibrosis in type 1-like diabetic rats. Phytother. Res. 31, 638–646. 10.1002/ptr.5777 28176375

[B53] Lopez B. Querejeta R. Gonzalez A. Sanchez E. Larman M. Diez J. (2004). Effects of loop diuretics on myocardial fibrosis and collagen type I turnover in chronic heart failure. J. Am. Coll. Cardiol. 43, 2028–2035. 10.1016/j.jacc.2003.12.052 15172408

[B54] Luo X. Deng Q. Xue Y. Zhang T. Wu Z. Peng H. (2021b). Anti-fibrosis effects of magnesium lithospermate B in experimental pulmonary fibrosis: By inhibiting TGF-βri/smad signaling. Molecules 26, 1715. 10.3390/molecules26061715 33808650 PMC8003516

[B55] Luo X. Deng Q. Xue Y. Zhang T. Wu Z. Peng H. (2021a). Anti-fibrosis effects of magnesium lithospermate B in experimental pulmonary fibrosis: By inhibiting TGF-βri/smad signaling. Molecules 26, 1715. 10.3390/molecules26061715 33808650 PMC8003516

[B56] Ma S. Yang D. Wang K. Tang B. Li D. Yang Y. (2012). Cryptotanshinone attenuates isoprenaline-induced cardiac fibrosis in mice associated with upregulation and activation of matrix metalloproteinase-2. Mol. Med. Rep. 6, 145–150. 10.3892/mmr.2012.866 22505122

[B57] Ma S. Zhang D. Lou H. Sun L. Ji J. (2016). Evaluation of the anti-inflammatory activities of tanshinones isolated from Salvia miltiorrhiza var. alba roots in THP-1 macrophages. J. Ethnopharmacol. 188, 193–199. 10.1016/j.jep.2016.05.018 27178632

[B58] Ma X. H. Ma Y. Tang J. F. He Y. L. Liu Y. C. Ma X. J. (2015). The biosynthetic pathways of tanshinones and phenolic acids in salvia miltiorrhiza. Molecules 20, 16235–16254. 10.3390/molecules200916235 26370949 PMC6332233

[B59] Ma Y. Li H. Yue Z. Guo J. Xu S. Xu J. (2014). Cryptotanshinone attenuates cardiac fibrosis via downregulation of COX-2, NOX-2, and NOX-4. J. Cardiovasc. Pharmacol. 64, 28–37. 10.1097/FJC.0000000000000086 24621647

[B60] Maki T. Kawahara Y. Tanonaka K. Yagi A. Takeo S. (2002). Effects of tanshinone VI on the hypertrophy of cardiac myocytes and fibrosis of cardiac fibroblasts of neonatal rats. Planta Med. 68, 1103–1107. 10.1055/s-2002-36337 12494338

[B61] Nan J. X. Park E. J. Kang H. C. Park P. H. Kim J. Y. Sohn D. H. (2001). Anti-fibrotic effects of a hot-water extract from Salvia miltiorrhiza roots on liver fibrosis induced by biliary obstruction in rats. J. Pharm. Pharmacol. 53, 197–204. 10.1211/0022357011775406 11273016

[B62] Noble P. W. Barkauskas C. E. Jiang D. (2012). Pulmonary fibrosis: Patterns and perpetrators. J. Clin. Invest. 122, 2756–2762. 10.1172/JCI60323 22850886 PMC3408732

[B63] Pan Y. Fu H. Kong Q. Xiao Y. Shou Q. Chen H. (2014). Prevention of pulmonary fibrosis with salvianolic acid a by inducing fibroblast cell cycle arrest and promoting apoptosis. J. Ethnopharmacol. 155, 1589–1596. 10.1016/j.jep.2014.07.049 25102244

[B64] Pang H. Wu L. Tang Y. Zhou G. Qu C. Duan J. A. (2016). Chemical Analysis of the Herbal Medicine Salviae miltiorrhizae Radix et Rhizoma (Danshen). Molecules 21, 51. 10.3390/molecules21010051 26742026 PMC6273254

[B65] Parajuli D. R. Park E. J. Che X. H. Jiang W. Y. Kim Y. C. Sohn D. H. (2013). PF2401-SF, standardized fraction of Salvia miltiorrhiza, induces apoptosis of activated hepatic stellate cells *in vitro* and *in vivo* . Molecules 18, 2122–2134. 10.3390/molecules18022122 23389256 PMC6270605

[B66] Parajuli D. R. Zhao Y. Z. Jin H. Chi J. H. Li S. Y. Kim Y. C. (2015). Anti-fibrotic effect of PF2401-SF, a standardized fraction of Salvia miltiorrhiza, in thioacetamide-induced experimental rats liver fibrosis. Arch. Pharm. Res. 38, 549–555. 10.1007/s12272-014-0425-2 25005065

[B67] Peng L. Y. An L. Sun N. Y. Ma Y. Zhang X. W. Liu W. H. (2019). Salvia miltiorrhiza restrains reactive oxygen species-associated pulmonary fibrosis via targeting nrf2-nox4 redox balance. Am. J. Chin. Med. 47, 1113–1131. 10.1142/S0192415X19500575 31352786

[B68] Peng R. Wang S. Wang R. Wang Y. Wu Y. Yuan Y. (2017). Antifibrotic effects of tanshinol in experimental hepatic fibrosis by targeting PI3K/AKT/mTOR/p70S6K1 signaling pathways. Discov. Med. 23, 81–94. 28371611

[B69] Peng Y. Yang T. Huang K. Shen L. Tao Y. Liu C. (2018). Salvia miltiorrhiza ameliorates liver fibrosis by activating hepatic natural killer cells *in vivo* and *in vitro* . Front. Pharmacol. 9, 762. 10.3389/fphar.2018.00762 30061833 PMC6054956

[B70] Qiu H. Liu W. Lan T. Pan W. Chen X. Wu H. (2018b). Salvianolate reduces atrial fibrillation through suppressing atrial interstitial fibrosis by inhibiting TGF-β1/Smad2/3 and TXNIP/NLRP3 inflammasome signaling pathways in post-MI rats. Phytomedicine 51, 255–265. 10.1016/j.phymed.2018.09.238 30466624

[B71] Qiu H. Liu W. Lan T. Pan W. Chen X. Wu H. (2018a). Salvianolate reduces atrial fibrillation through suppressing atrial interstitial fibrosis by inhibiting TGF-β1/Smad2/3 and TXNIP/NLRP3 inflammasome signaling pathways in post-MI rats. Phytomedicine 51, 255–265. 10.1016/j.phymed.2018.09.238 30466624

[B72] Schuppan D. Kim Y. O. (2013). Evolving therapies for liver fibrosis. J. Clin. Invest. 123, 1887–1901. 10.1172/JCI66028 23635787 PMC3635731

[B73] Shi M. J. Yan X. L. Dong B. S. Yang W. N. Su S. B. Zhang H. (2020). A network pharmacology approach to investigating the mechanism of Tanshinone IIA for the treatment of liver fibrosis. J. Ethnopharmacol. 253, 112689. 10.1016/j.jep.2020.112689 32101775

[B74] Su C. Y. Ming Q. L. Rahman K. Han T. Qin L. P. (2015). Salvia miltiorrhiza: Traditional medicinal uses, chemistry, and pharmacology. Chin. J. Nat. Med. 13, 163–182. 10.1016/S1875-5364(15)30002-9 25835361

[B75] Sun J. Huang S. H. Tan B. K. Whiteman M. Zhu Y. C. Wu Y. J. (2005). Effects of purified herbal extract of Salvia miltiorrhiza on ischemic rat myocardium after acute myocardial infarction. Life Sci. 76, 2849–2860. 10.1016/j.lfs.2004.11.016 15808885

[B76] Tian S. Chen M. Wang B. Han Y. Shang H. Chen J. (2021). Salvianolic acid B blocks hepatic stellate cell activation via FGF19/FGFR4 signaling. Ann. Hepatol. 20, 100259. 10.1016/j.aohep.2020.07.013 32980439

[B77] Tsochatzis E. A. Bosch J. Burroughs A. K. (2014). Liver cirrhosis. Lancet 383, 1749–1761. 10.1016/S0140-6736(14)60121-5 24480518

[B78] Wan Y. J. Wang Y. H. Guo Q. Jiang Y. Tu P. F. Zeng K. W. (2021). Protocatechualdehyde protects oxygen-glucose deprivation/reoxygenation-induced myocardial injury via inhibiting PERK/ATF6α/IRE1α pathway. Eur. J. Pharmacol. 891, 173723. 10.1016/j.ejphar.2020.173723 33159933

[B79] Wang C. Luo H. Xu Y. Tao L. Chang C. Shen X. (2018a). Salvianolic acid B-alleviated angiotensin II induces cardiac fibrosis by suppressing NF-κB pathway *in vitro* . Med. Sci. Monit. 24, 7654–7664. 10.12659/MSM.908936 30365482 PMC6215385

[B80] Wang L. Yu J. Fordjour P. A. Xing X. Gao H. Li Y. (2017). Danshen injection prevents heart failure by attenuating post-infarct remodeling. J. Ethnopharmacol. 205, 22–32. 10.1016/j.jep.2017.04.027 28465251

[B81] Wang R. Wang J. Song F. Li S. Yuan Y. (2018b). Tanshinol ameliorates CCl(4)-induced liver fibrosis in rats through the regulation of Nrf2/HO-1 and NF-κB/IκBα signaling pathway. Drug Des. devel. Ther. 12, 1281–1292. 10.2147/DDDT.S159546 PMC5961642 29844659

[B82] Wang R. Yu X. Y. Guo Z. Y. Wang Y. J. Wu Y. Yuan Y. F. (2012). Inhibitory effects of salvianolic acid B on CCl(4)-induced hepatic fibrosis through regulating NF-κB/IκBα signaling. J. Ethnopharmacol. 144, 592–598. 10.1016/j.jep.2012.09.048 23041223

[B83] Wang Y. Peng H. Shen Y. Zhao R. Huang L. (2013). The profiling of bioactive ingredients of differently aged Salvia miltiorrhiza roots. Microsc. Res. Tech. 76, 947–954. 10.1002/jemt.22253 23839871

[B84] Wu C. Chen W. Ding H. Li D. Wen G. Zhang C. (2019). Salvianolic acid B exerts anti-liver fibrosis effects via inhibition of MAPK-mediated phospho-Smad2/3 at linker regions *in vivo* and *in vitro* . Life Sci. 239, 116881. 10.1016/j.lfs.2019.116881 31678285

[B85] Wynn T. A. (2004). Fibrotic disease and the T(H)1/T(H)2 paradigm. Nat. Rev. Immunol. 4, 583–594. 10.1038/nri1412 15286725 PMC2702150

[B86] Wynn T. A. (2011). Integrating mechanisms of pulmonary fibrosis. J. Exp. Med. 208, 1339–1350. 10.1084/jem.20110551 21727191 PMC3136685

[B87] Wynn T. A. Ramalingam T. R. (2012). Mechanisms of fibrosis: Therapeutic translation for fibrotic disease. Nat. Med. 18, 1028–1040. 10.1038/nm.2807 22772564 PMC3405917

[B88] Xd M. E. Cao Y. F. Che Y. Y. Li J. Shang Z. P. Zhao W. J. (2019). Danshen: A phytochemical and pharmacological overview. Chin. J. Nat. Med. 17, 59–80. 10.1016/S1875-5364(19)30010-X 30704625

[B89] Xu L. Shen P. Bi Y. Chen J. Xiao Z. Zhang X. (2016). Danshen injection ameliorates STZ-induced diabetic nephropathy in association with suppression of oxidative stress, pro-inflammatory factors and fibrosis. Int. Immunopharmacol. 38, 385–394. 10.1016/j.intimp.2016.06.024 27355131

[B90] Xu S. He L. Ding K. Zhang L. Xu X. Wang S. (2020). Tanshinone IIA ameliorates streptozotocin-induced diabetic nephropathy, partly by attenuating PERK pathway-induced fibrosis. Drug Des. devel. Ther. 14, 5773–5782. 10.2147/DDDT.S257734 PMC7780857 33408464

[B91] Yang J. Li J. Tan R. He X. Lin X. Zhong X. (2021). Protocatechualdehyde attenuates obstructive nephropathy through inhibiting lncRNA9884 induced inflammation. Phytother. Res. 35, 1521–1533. 10.1002/ptr.6919 33118280

[B92] Yang T. Shen D. P. Wang Q. L. Tao Y. Y. Liu C. H. (2013). Investigation of the absorbed and metabolized components of Danshen from Fuzheng Huayu recipe and study on the anti-hepatic fibrosis effects of these components. J. Ethnopharmacol. 148, 691–700. 10.1016/j.jep.2013.05.031 23707207

[B93] Yang Y. Yang S. Chen M. Zhang X. Zou Y. Zhang X. (2008). Compound Astragalus and Salvia miltiorrhiza Extract exerts anti-fibrosis by mediating TGF-beta/Smad signaling in myofibroblasts. J. Ethnopharmacol. 118, 264–270. 10.1016/j.jep.2008.04.012 18502066

[B94] Yin D. Yin J. Yang Y. Chen S. Gao X. (2014). Renoprotection of Danshen Injection on streptozotocin-induced diabetic rats, associated with tubular function and structure. J. Ethnopharmacol. 151, 667–674. 10.1016/j.jep.2013.11.025 24269771

[B95] Yin Q. Lu H. Bai Y. Tian A. Yang Q. Wu J. (2015). A metabolite of Danshen formulae attenuates cardiac fibrosis induced by isoprenaline, via a NOX2/ROS/p38 pathway. Br. J. Pharmacol. 172, 5573–5585. 10.1111/bph.13133 25766073 PMC4667860

[B96] Zeng J. Bao X. (2021b). Tanshinone IIA attenuates high glucose-induced epithelial-to-mesenchymal transition in HK-2 cells through VDR/Wnt/β-catenin signaling pathway. Folia histochem. Cytobiol. 59, 259–270. 10.5603/FHC.a2021.0025 34852178

[B97] Zeng J. Bao X. (2021a). Tanshinone IIA attenuates high glucose-induced epithelial-to-mesenchymal transition in HK-2 cells through VDR/Wnt/β-catenin signaling pathway. Folia histochem. Cytobiol. 59, 259–270. 10.5603/FHC.a2021.0025 34852178

[B98] Zhang L. Wu T. Chen J. M. Yang L. L. Song H. Y. Ji G. (2012). Danshensu inhibits acetaldehyde-induced proliferation and activation of hepatic stellate cell-T6. Zhong Xi Yi Jie He Xue Bao 10, 1155–1161. 10.3736/jcim20121013 23073200

[B99] Zhang W. Ping J. Zhou Y. Chen G. Xu L. (2019a). Salvianolic acid B inhibits activation of human primary hepatic stellate cells through downregulation of the myocyte enhancer factor 2 signaling pathway. Front. Pharmacol. 10, 322. 10.3389/fphar.2019.00322 31031620 PMC6470251

[B100] Zhang Y. Li X. Wang Z. (2013). Diversity evaluation of Salvia miltiorrhiza using ISSR markers. Biochem. Genet. 51, 707–721. 10.1007/s10528-013-9600-2 23712760

[B101] Zhang Y. Lu W. Zhang X. Lu J. Xu S. Chen S. (2019b). Cryptotanshinone protects against pulmonary fibrosis through inhibiting Smad and STAT3 signaling pathways. Pharmacol. Res. 147, 104307. 10.1016/j.phrs.2019.104307 31181334

[B102] Zhang Y. Wang H. Cui L. Zhang Y. Liu Y. Chu X. (2015). Continuing treatment with Salvia miltiorrhiza injection attenuates myocardial fibrosis in chronic iron-overloaded mice. PLoS One 10, e0124061. 10.1371/journal.pone.0124061 25850001 PMC4388639

[B103] Zhao Q. Pan Y. L. Dou H. T. Hua J. H. Fu X. X. Wang J. H. (2016a). Effect of different locations and genotypes on yield and accumulation of bioactive constituents in salvia miltiorrhiza. Zhong Yao Cai 39, 1935–1939. 30207648

[B104] Zhao Q. Song Z. Fang X. Pan Y. Guo L. Liu T. (2016b). Effect of genotype and environment on salvia miltiorrhiza roots using LC/MS-Based metabolomics. Molecules 21, 414. 10.3390/molecules21040414 27023512 PMC6273704

